# Manifestation of Guillain–Barre Syndrome in a Case of Monkeypox Virus Infection: A Rare Case Report

**DOI:** 10.1155/2023/2426659

**Published:** 2023-09-11

**Authors:** Tariq Abdul Hamid, Nayab Mustafa, Almas Zulfiquar Parkar, Sherin Marina Varghese, Ossama Sayedahmed, Mohammed Ghanaim

**Affiliations:** ^1^Department of Urology, Dubai Hospital, Dubai Health Authority, Dubai, UAE; ^2^Dubai Health Authority, Dubai, UAE; ^3^Department of Anaesthesia, Dubai Hospital, Dubai Health Authority, Dubai, UAE; ^4^Emergency Department, Dubai Hospital, Dubai Health Authority, Dubai, UAE

## Abstract

Monkeypox virus (MPXV) is one of the rare zoonotic infections caused by orthopoxvirus. MPXV has recently been an evolving threat to public health with its contagious human-to-human transmission. Various presentations of MPXV infection have been reported ranging from generalised symptoms such as fever, chills, body aches, and swollen lymph nodes to dermatological presentations. Neurological manifestations that have been reported include headaches, myalgia, seizures, and even mood disturbances. Postinfectious complications such as encephalitis, vision problems, and skin infections have also been noticed. Guillain–Barre Syndrome (GBS) is an acquired acute inflammatory polyradiculoneuropathy characterized by progressive, symmetrical, proximal, and distal tingling and weakness. Although various microorganisms are known to cause GBS and have been linked to the smallpox vaccine, they are rarely linked to MPXV disease. In this report, we describe a case of a confirmed monkeypox infection in a patient presenting with Guillain-Barre Syndrome.

## 1. Introduction

Monkeypox virus (MPXV) infection is a zoonotic disease caused by the monkeypox virus, originating from the same family of viruses that causes small pox [1]. MPXV cases have commonly been reported in central and western Africa but its emergence internationally has been escalating and has resulted in a worldwide outbreak beginning in May 2022 and affecting over 86,000 people in one year [2, 3]. Amongst these, almost 100 deaths have been reported globally by March 2023 [1]. The drastic increase in the number of cases led to MPXV being declared a global emergency. It is believed that the absence of a smallpox vaccination regime and decreasing immunity against smallpox could be one of the possible causes of the MPXV outbreak [4]. Concurrently, it was also found that the contagion was interhuman, particularly through sexual contact [5].

The incubation period of MPXV infection is about 5–21 days with symptoms including fever (between 38.5°C and 40.5°C) followed by a centrifugal rash, headache, and myalgia. In addition, the presence of maxillary, cervical, or inguinal lymphadenopathy is a distinguishing feature of the infection [4]. However, varying presentations have been noticed in the current outbreak. Atypical presentations including skin lesions without a prodrome, predominant anogenital or oropharyngeal lesions, and proctitis have been observed. Ocular manifestations reported include conjunctivitis and corneal lesions leading to scarring and visual loss [2]. Neurological manifestations have also been reported ranging from headaches, myalgia, seizures, and even mood disturbances [5]. In additionally, seizures and encephalitis are known to occur rarely [2].

In terms of preventive measures, it was found that smallpox vaccines exhibit cross-protective activity against monkeypox. Two vaccines have been approved by the US Food and Drug Administration (FDA), a second-generation live VACV vaccine, ACAM2000, and an attenuated third-generation vaccine based on modified vaccinia Ankara (MVA), JYNNEOS. Side effects of administration include myocarditis and pericarditis with higher risks in individuals with eczema and those pregnant. Postexposure vaccination for up to 3 days is thought to be effective for disease prevention and for reduction of disease severity in an unvaccinated person [4].

## 2. Case Report

**Figure 1:**
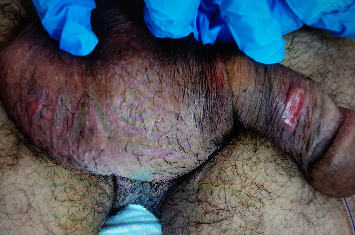
Monkeypox lesions noted at the genitalia including the scrotum and the penile shaft.

**Figure 2:**
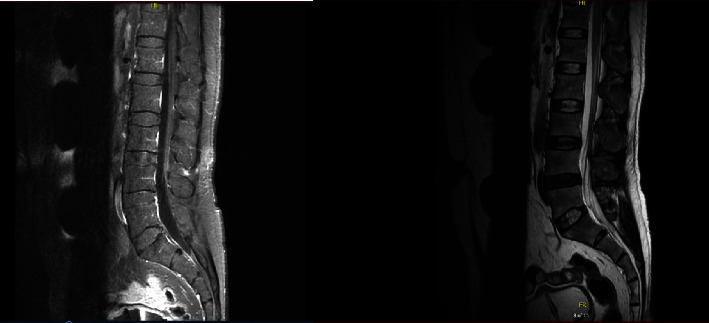
Mild enhancement of the cauda equina roots.

A 53-year-old heterosexual unmarried male construction worker weighing 75 kg presented to the emergency with an acute onset of bilateral lower limb weakness and difficulty in walking associated with the inability to pass urine for one day. The patient also reported having a vesicular rash over his head and external genitalia (Figure 1) along with a fever for two days following unprotected sexual intercourse. He was otherwise hemodynamically stable and oriented. He did not manifest any cranial nerve involvement, sensory symptoms, or any weakness in his upper limbs. Neurological examination revealed areflexia in the lower limbs, muscle power of 0-1/5, and down-going plantar reflexes. The patient was then referred to the urology department for catheterization, and cauda equina syndrome was ruled out. The patient underwent serological screening for varicella zoster, hepatitis B, hepatitis C, and HIV and meningitis/encephalitis multiplex PCR testing for other viral aetiologies including cytomegalovirus, respectively, all of which were negative. Due to the recent outbreak of MPXV, samples were also taken from the genital lesions for the monkeypox DNA PCR test which resulted positive. A computed tomography (CT) scan of the brain was done, which was unremarkable, and a lumbar puncture carried out resulted in elevated proteins of 106 mg/dl (15–45 mg/dl) and glucose of 58 mg/dl (40–76 mg/dl) in cerebrospinal fluid (CSF) including CSF cultures which showed no obvious pathogen including mycobacterium tuberculosis. Other labs such as urine culture, WBC, and basic biochemistry were normal except for creatine phosphokinase (CPK), which was 450 U/L (0–190 U/L). Thus, neurologists were consulted who advised magnetic resonance imaging (MRI) of the thoracolumbar spine which showed no signs of compressive myelopathy, but mild enhancement of a few cauda equina nerve roots was noted (Figure 2). Considering the acute bilateral limb weakness, with lumbar puncture results and positive DNA PCR results of monkeypox, a diagnosis of Guillain–Barre Syndrome (GBS) as a sequel to monkeypox infection was made.

Neurologists further suggested IV immunoglobulins (IVIG) 0.4 g/kg as a treatment given along with close monitoring of the patient with serial arterial blood gas monitoring for any desaturation. The patient was kept on high-flow oxygen, maintaining a saturation of 94-95%, and was transferred to the neurology ward for further care. The vesicular rashes were managed by regular sterile dressings and antibiotics. On Day 2 of the hospital stay, the patient had a saturation drop to 90% and was complaining of weakness during mastication and neck weakness and thus was continued on high-flow oxygen and an IVIG course of 5 days. A nasogastric tube was inserted due to suspected bulbar weakness for enteral feeding. By day 7, the patient reported improvement in his lower limb weakness increasing to 3/5, oxygen saturation was stabilized at 97-98%; and gradually an improvement in his feeding habits was noticed with good oral intake. However, the patient complained of neuropathic pain in his lower limbs, for which gabapentin was prescribed, and was advised for further inpatient monitoring. Unfortunately, he refused due to financial reasons and was discharged in stable condition with the advice of a neurology follow-up.

## 3. Discussion

The current unprecedented monkeypox outbreak has spread to over 96 countries, and cases have commonly been reported in men of homosexual orientation. Neurological manifestations of MPXV diversely present from mildly severe symptoms such as headaches, myalgia, neuropathic pain, and photophobia to more serious complications such as seizures and encephalitis [5, 6]. The most common neurological symptom so far has been reported as a generalised or frontal, prodromal headache. Furthermore, a not yet peer-reviewed preprint meta-analysis also suggests that headache constitutes nearly 53.8% of the most commonly reported neurologic presentations in MPXV disease whereas only 2% of the cases have reported seizure, confusion, and encephalitis as rarer outcomes [7, 8]. Patients have also experienced mood disturbances such as depression, anxiety, and suicide [9].

The neuroinvasive potential of MPXV has been known to be rare, but it can still be present in immunocompromised individuals and is particularly seen in cases with HIV infections [7]. Amidst 40 MPXV-related hospitalizations in Nigeria, two cases, including a 28-day-old baby and a 43-year-old man with HIV, were suspected to have encephalitis and seizures [10]. Our patient, on the other hand, presented primarily with bilateral lower limb weakness and difficulties in walking with no previous medical illnesses. A positive correlation in the neuroinvasive effects of MPXV has also been seen in those patients who were never vaccinated against smallpox as in the case report of a 3-year-old unvaccinated girl who was admitted with MPXV-associated encephalitis and died after being comatose for two days [5]. However, our patient reported an unclear history of both smallpox disease and prior vaccination. Neuroinvasive properties of the virus have also presented as nonspecific encephalopathy as well as postinfectious acute disseminated encephalomyelitis (ADEM) in about three-quarters of cases [11, 12]. This has also been reported in the U.S.A. in 2022 in two MPXV-associated acute disseminated encephalomyelitis cases which have been reported in previously healthy, young, gay men [13]. Meanwhile, our patient was fully oriented on presentation and throughout his hospital stay had no signs of confusion or meningeal irritation.

GBS is known to be an acute immune-mediated polyneuropathy. Two-thirds of the patients report a bacterial or viral prodrome with the most common organism being Campylobacter jejuni, before developing symptoms of GBS [14]. Whilst a few case reports can be found describing various types of rashes and GBS, GBS has rarely been reported as a complication of MPXV disease. Daher reported a 36-year-old man with an asymptomatic maculopapular rash and limb weakness subsequently diagnosed as GBS [14]. In contrast, Khalifa and Zakaria reported an 11-year-old boy diagnosed with GBS and SARS-CoV-2, who further developed a nonpruritic morbilliform rash on his palmar surfaces [15]. However, it should be noted that studies have shown smallpox vaccines, such as ACAM2000, which have been linked to serious neurological adverse effects, including Guillain–Barre Syndrome (GBS) [16].

## 4. Conclusion

The neuroinvasive propensity of MPXV is still quite understudied, and it is essential that such atypical neurological presentations of MPXV should be expected as potential complications. Neurological signs manifested by patients positive for monkeypox virus infection varied and at times may present as an acute onset of limb weakness or bulbar weakness, which is potentially life-threatening. Early diagnosis and management are essential for improving the outcome. Patients who have been exposed to monkeypox or are at higher risk of being exposed may be vaccinated against monkeypox to reduce the chance of disease and can consider contact precautionary measures to reduce their risk of exposure to MPXV.

## Data Availability

The authors confirm that the data supporting the findings of this study are included within the article. Raw data that support the findings of this study are available from the corresponding author upon reasonable request.
